# Comparison of Commercially Available and Laboratory-Developed Assays for *In Vitro* Detection of SARS-CoV-2 in Clinical Laboratories

**DOI:** 10.1128/JCM.00821-20

**Published:** 2020-07-23

**Authors:** Joshua A. Lieberman, Gregory Pepper, Samia N. Naccache, Meei-Li Huang, Keith R. Jerome, Alexander L. Greninger

**Affiliations:** a Department of Laboratory Medicine, University of Washington Medical Center, Seattle, Washington, USA; b LabCorp Seattle, Department of Microbiology, Seattle, Washington, USA; c Vaccine and Infectious Disease Division, Fred Hutchinson Cancer Research Center, Seattle, Washington, USA; Boston Children’s Hospital

**Keywords:** COVID, COVID-19, SARS, SARS-CoV-2, comparison, coronavirus, qPCR

## Abstract

Multiple laboratory-developed tests (LDTs) and commercially available assays have emerged to meet diagnostic needs related to the severe acute respiratory syndrome coronavirus 2 (SARS-CoV-2) pandemic. To date, there is limited comparison data for these different testing platforms. We compared the analytical performance of a LDT developed in our clinical laboratory based on CDC primer sets and four commercially available, FDA emergency use authorized assays for SARS-CoV-2 (Cepheid, DiaSorin, Hologic Panther, and Roche Cobas) on a total of 169 nasopharyngeal swabs. The LDT and Cepheid Xpert Xpress SARS-CoV-2 assays were the most sensitive assays for SARS-CoV-2 with 100% agreement across specimens. The Hologic Panther Fusion, DiaSorin Simplexa, and Roche Cobas 6800 failed to detect positive specimens only near the limit of detection of our CDC-based LDT assay. All assays were 100% specific, using our CDC-based LDT as the gold standard. Our results provide initial test performance characteristics for SARS-CoV-2 reverse transcription-PCR (RT-PCR) and highlight the importance of having multiple viral detection testing platforms available in a public health emergency.

## Abstract (precis)

Multiple laboratory-developed tests (LDTs) and commercially available assays have emerged to meet diagnostic needs related to the severe acute respiratory syndrome coronavirus 2 (SARS-CoV-2) pandemic. To date, there is limited comparison data for these different testing platforms. We compared the analytical performance of a LDT developed in our clinical laboratory based on CDC primer sets and four commercially available, FDA emergency use authorized assays for SARS-CoV-2 (Cepheid, DiaSorin, Hologic Panther, and Roche Cobas) on a total of 169 nasopharyngeal swabs.

## INTRODUCTION

Since the first infection with severe acute respiratory syndrome coronavirus 2 (SARS-CoV-2) was detected in the United States in January 2020 (1), there has been an exponential growth in cases and deaths (2). At the time of this writing, the U.S. case count exceeds 600,000 with more than 30,000 deaths and considerable geographic heterogeneity (2, 3). Despite social distancing policies, the outbreak of coronavirus disease 2019 (COVID-19), the disease caused by SARS-CoV-2, continues to grow and threatens to overwhelm hospital systems in multiple states (2).

The explosion of COVID-19 cases in the United States has highlighted the critical role diagnostic testing plays in medical and public health decision-making in containing and mitigating the SARS-CoV-2 pandemic. Reliable test results enable appropriate utilization of scarce hospital resources, including personal protective equipment (PPE) and negative-pressure isolation rooms, as well as public health resources for contact tracing or isolation decision-making (4). In rapid succession in March 2020, multiple assays have become available, including both FDA emergency use authorization (EUA) test platforms and laboratory-developed tests (LDTs) for use in high-complexity clinical laboratories. To solve supply chain difficulties, clinical laboratories have had to implement multiple assays using scarce reagent resources, rendering thorough comparisons challenging. A clear understanding of the analytical parameters of these options is important to help guide assay selection by clinical laboratories when supply chain considerations subside (4).

Reverse transcription-PCR (RT-PCR) is the mainstay of SARS-CoV-2 detection *in vitro* (5). FDA EUA authorized assays for SARS-CoV-2 have mostly targeted two loci of the positive-sense, single-stranded RNA virus by real-time RT-PCR and are reported qualitatively. Our laboratory has recently reported that the CDC N2 and WHO E-gene primer/probe sets are among the most sensitive and have detected no false-positive results in our LDTs (6). FDA EUA authorized platforms use a variety of different primer/probe sets, resulting in the potential for differing analytical sensitivities. In addition to differing analytical sensitivities, commercially available platforms have important operational differences, including integrated sample extraction, run time, random access, and acceptable sample types.

Here, the performances of one LDT-EUA assay developed in our clinical laboratory and four FDA-EUA cleared assays were evaluated for detection of SARS-CoV-2. The FDA-EUA cleared assays included were Hologic Panther Fusion (both research use only [RUO] and EUA versions; EUA version performed at two study sites), DiaSorin Simplexa COVID-19 Direct (EUA), Cepheid Xpert Xpress SARS-CoV-2 (EUA), and Roche Cobas 6800 (EUA). The test performance characteristics of each RT-PCR were determined compared to those of our reference LDT assay.

## MATERIALS AND METHODS

### Specimen collection and consensus panel selection.

Nasopharyngeal (NP) swabs (*n *= 169) were collected from patient specimens submitted to the University of Washington Medical Center laboratories for clinical diagnostic testing. LDT performance was validated based on detection of 20 of 20 positive specimens sent by the Washington State Public Health Laboratory in early March. Residual clinical samples were used for validation/verification of each subsequent instrument, including a common panel of 26 specimens (12 positive, 1 inconclusive, and 13 negative) tested at the University of Washington (UW) by the UW CDC EUA-based LDT (CDC LDT), DiaSorin Simplexa (positive specimens only), Roche Cobas 6800, and tested at LabCorp Seattle on the Cepheid Xpert Xpress, and Panther Fusion (12 positive specemens only). Additional residual (*n *= 115) specimens were tested at the UW on individual assays and compared to the reference method (LDT): Panther Fusion (RUO), *n *= 36; Panther Fusion (EUA)-UW, *n *= 20; DiaSorin Simplexa (EUA), *n *= 19; Cobas 6800, *n *= 40. Finally, 28 specimens were used to compare the SARS-CoV-2 assay on the UW Panther Fusion with the DiaSorin Simplexa assay. All same-sample comparisons were performed on specimens stored at 4°C for less than 72 h with no freeze-thaws. Inconclusive results (one of two targets detected) were considered positive due to the high specificity of all assays and limited cross-reactivity seen for SARS-CoV-2 primer sets. This work was approved under a consent waiver from the University of Washington Institutional Review Board.

### Sample processing.

For the UW CDC LDT, total nucleic acid (NA) was extracted from 200 μl of viral transport medium (VTM) on the Roche MP96 and eluted in 50 μl of elution buffer. Real-time RT-PCR was set up on 5 μl of eluate using the CDC N1, N2, and RP (or Exo internal control) primers and run on ABI 7500 real-time PCR instruments as reported previously (6). For the Hologic Panther Fusion, 500 μl of VTM was transferred to lysis buffer in manufacturer-provided tubes and loaded directly on the instrument. For the DiaSorin Simplexa and Cepheid Xpert Xpress, 50 μl or 300 μl of VTM sample, respectively, was loaded directly into the reaction cartridge with integrated sample process. For the Roche Cobas 6800, 600 μl of specimen VTM was added to a barcoded secondary tube (12 by 75 mm) and loaded directly on the instrument.

## RESULTS

### Panther Fusion SARS-CoV-2.

The Panther Fusion SARS-CoV-2 assay was tested first as research use only (RUO) reagents (*n *= 36) and tested again following FDA authorization (*n *= 20). Both Panther Fusion RUO and EUA assays were slightly less sensitive than the CDC-based LDT, missing one positive/inconclusive sample in each sample set (Tables 1 and 2). One additional specimen was initially negative with the RUO reagents but was detected upon repeat with the Panther Fusion EUA assay. Discordant specimens were either inconclusive (one target of two detected) or had high average threshold cycles (*C_T_*) (>37) by the CDC LDT test. All 29 negative specimens generated “Not detected” results by the Hologic Panther Fusion SARS-CoV-2 assay.

**TABLE 1:** CDC-based LDT versus Hologic Panther Fusion RUO^*a*^

Sample ID	*C_T_* found using the following test and primer or gene:		
	UW IDT		Panther fusion SARS-CoV-2 (RUO), Orf1ab/2ab
	N1	N2	
56	27.2	27.8	23.2
07	26.2	25.5	22.8
46	23.9	25.8	21.4
81	23.8	24	25.5
40	23.8	23.9	23.7
66	17.7	17.1	19.7
26	24.9	24.6	27
85	35.98	35.8	33.5
82	29.1	29.7	29.6
37	23.1	22.3	22
70	29.7	28.9	30.5
29	29.4	28.2	26.3
68	29.4	28	28.3
04	25.9	25.4	24.9
95	35	37	35.3
55	39.2	39	NDET
14	36.3	NDET	35.5

a All 19 CDC LDT-negative specimens were negative by Hologic Panther Fusion EUA. Abbreviations: ID, identifier; NDET, not detected.

**TABLE 2:** CDC-based LDT versus Hologic Panther Fusion EUA^*a*^

Sample ID	*C_T_* found using the following test and primer or gene:		
	UW IDT		Panther fusion SARS-CoV-2 (EUA), Orf1ab/2ab
	N1	N2	
100	30	30.4	29
67	20.3	20.8	19.3
76	27.1	27.6	31.1
17	23.4	23.6	20.5
02	18.1	17.2	19.3
59	29.4	29.8	31.7
97	18.4	17	18.9
52	16.8	15.9	16.9
90	36.1	38.4	NDET/38.1
79	38.4	NDET	NDET/NDET

a All 10 CDC LDT-negative specimens were negative by Hologic Panther Fusion EUA. NDET, not detected.

### DiaSorin Simplexa SARS-CoV-2.

We next compared the DiaSorin Simplexa SARS-CoV-2 assay to our CDC-based LDT. All 19 specimens (11 positives and 8 negatives) demonstrated complete concordance between the two platforms (Table 3) with lower *C_T_*s recovered by the DiaSorin assay compared to the LDT on all specimens (average *C_T_* difference of −2.1 [interquartile range {IQR}, −2.3 to −1.7]). When we compared SARS-CoV-2 detection on the DiaSorin Simplexa to the Hologic Panther Fusion, all 16 Hologic Panther Fusion positive specimens were detected by the DiaSorin Simplexa, while the DiaSorin Simplexa generated one additional positive result in the 12 specimens that were negative by the Hologic Panther Fusion (Table 4). This discordant specimen was detected by the CDC-based LDT with *C_T_*s of 36.8 (N1) and 35.8 (N2), confirming the DiaSorin Simplexa result.

**TABLE 3:** CDC-based LDT versus DiaSorin Simplexa EUA^*a*^

Sample ID	*C_T_* found using the following test and primer or gene:			
	UW IDT		UW DiaSorin	
	N1	N2	S gene	ORF1ab
1A Pos	27.9	27.6	25.2	25.6
2A Pos	18.6	19.1	18.0	18.1
3A Pos	27.8	27.5	25.2	26.0
4A Pos	28.2	27.6	25.8	26.5
5A Pos	31.6	31.3	28.5	29.0
6A Pos	33.9	34.6	31.0	31.6
7A Pos	31.1	31.5	28.8	29.2
1C Pos	34.1	33.9	31.5	32.1
2C Pos	33.7	34.6	32.7	32.4
3C Pos	32.4	32.3	29.8	30.4
4C Pos	34.9	34.8	32.7	33.6

a All eight negative specimens by UW LDT were negative by DiaSorin. Abbreviations: ID, identifier; Pos, positive.

**TABLE 4:** Hologic Panther Fusion EUA versus DiaSorin Simplex EUA^*a*^

Sample ID	*C_T_* found using the following test and gene:		
	Panther fusion SARS-CoV-2 (EUA), Orf1ab/2ab	DiaSorin	
		S gene	ORF1ab
65	35.2	37.2	32.9
38	33.6	33.7	34
83	31.5	28.5	27.7
13	30.7	27.2	27
39	30.1	30.7	29.2
10	29.6	29.1	28.1
56	28.7	27	26.4
31	26.5	24.4	22.8
33	25.4	24.8	24.4
60	23.3	21.1	20.5
92	22.5	23	23.4
98	21	18.1	17.3
40	18.2	15	14
25	17.9	16.1	15.6
13	16.7	15.1	14.3
52	15.6	13.2	12.3
42*	NDET	31.8	32.7

a Eleven of 12 specimens negative by Hologic Panther Fusion EUA were negative by DiaSorin. *, detected by UW CDC LDT (N1, 36.8; N2, 35.9).

### Roche Cobas SARS-CoV-2.

We next compared the Roche Cobas SARS-CoV-2 assay to our CDC LDT. All 20 negative specimens demonstrated complete concordance between the two platforms (Table 5). One of the 20 positive specimens was not detected by the Roche assay. This specimen had *C_T_*s of 38.0 (N1) and 37.4 (N2) in the LDT. Across the 20 positive specimens, *C_T_*s were only slightly higher on the Roche Cobas assay compared to the CDC-based LDT, with an average *C_T_* difference of 0.6 (IQR, −0.1 to 1.5.

**TABLE 5:** CDC-based LDT versus Cobas 6800 SARS-CoV-2^*a*^

Sample ID	*C_T_* found using the following test and primer or gene:			
	UW IDT		UW Cobas 6800	
	N1	N2	ORF1ab	E gene
136	26.7	27.6	26.9	27.2
560	16.4	16.3	19.0	19.6
578	23.8	24.9	25.4	26.3
757	23.4	24.2	24.7	25.0
982	20.3	20.9	21.9	22.2
853	20.4	21.5	21.6	21.5
998	14.6	15.6	15.9	16.3
334	20.2	21.4	21.5	21.9
571	18.0	18.9	17.9	18.2
108	35.5	35.0	31.8	34.7
188	36.4	35.7	35.4	37.2
599	24.7	25.6	26.7	27.1
995	25.2	26.4	26.4	26.8
336	29.1	29.5	31.1	31.6
866	31.3	31.4	31.1	32.0
232	36.3	36.4	32.3	35.2
323	28.6	28.7	29.5	30.7
309	14.3	15.4	14.5	14.8
277	19.7	21.4	20.4	20.5
018	38.0	37.4	NDET	NDET

a All 20 samples negative by UW LDT were negative by Cobas 6800. NDET, not detected.

### Five-way same-sample comparison, including Cepheid Xpert Xpress SARS-CoV-2 assay.

After performing the above pairwise comparisons, we next compared 26 specimens (13 positive, 13 negative) from another high-complexity hospital laboratory (LabCorp Seattle). All 26 specimens were also tested on the Cepheid Xpert Xpress SARS-CoV-2 assay (Table 6). All specimens with *C_T_*s of <35 on the CDC-based LDT were detected by all platforms, and all specimens not detected by the Cepheid Xpert were not detected by two other platforms examined (CDC LDT and Roche Cobas). One of 13 positive specimens was a presumptive positive on the Cepheid assay (E-gene *C_T_* of 42.6, N2 gene negative); upon repeat per package insert, the N2 gene was detected at a *C_T_* of 42.7 but the E gene was not detected, yielding a positive result. The CDC LDT demonstrated 100% concordance with the Cepheid Xpert Xpress, also detecting the extremely low viral load specimen above as an inconclusive (N1 *C_T_* of 37.4, N2 not detected). No other assay detected SARS-CoV-2 RNA in this specimen. In addition, the DiaSorin Simplexa failed to detect a positive specimen that on repeat was detected only by the ORF1ab primer set.

**TABLE 6:** Same-sample comparison of five testing platforms for SARS-CoV-2

Sample ID^*a*^	*C_T_* found using the following test platform(s) and primer or gene^*b*^:								
	UW IDT		UW DiaSorin		UW Cobas 6800		LabCorp Seattle		
							Xpert Xpress Sars-CoV2		Panther fusion SARS-CoV-2
	N1	N2	S gene	ORF1ab	ORF1ab	E gene	E gene	N2	Orf1ab/2ab
Neg 01	NDET	NDET	nd	nd	NDET	NDET	NDET	NDET	nd
Neg 02	NDET	NDET	nd	nd	NDET	NDET	NDET	NDET	nd
Neg 03	NDET	NDET	nd	nd	NDET	NDET	NDET	NDET	nd
Neg 04	NDET	NDET	nd	nd	NDET	NDET	NDET	NDET	nd
Neg 05	NDET	NDET	nd	nd	NDET	NDET	NDET	NDET	nd
Neg 06	NDET	NDET	nd	nd	NDET	NDET	NDET	NDET	nd
Neg 07	NDET	NDET	nd	nd	NDET	NDET	NDET	NDET	nd
Neg 08	NDET	NDET	nd	nd	NDET	NDET	NDET	NDET	nd
Neg 09	NDET	NDET	nd	nd	NDET	NDET	NDET	NDET	nd
Neg 10	NDET	NDET	nd	nd	NDET	NDET	NDET	NDET	nd
Neg 11	NDET	NDET	nd	nd	NDET	NDET	NDET	NDET	nd
Neg 12	NDET	NDET	nd	nd	NDET	NDET	NDET	NDET	nd
Neg 13	NDET	NDET	nd	nd	NDET	NDET	NDET	NDET	nd
Pos 01	30.7	30.2	29.2	30	30.5	31.1	31.7	33.8	31
Pos 02	28.5	28.7	27.2	28	29.6	30.5	29.2	31.6	29.7
Pos 03	28.6	28.8	27.3	28.4	30.4	32.2	28.7	31.4	31.2
Pos 04	25.2	24.4	22.4	23.8	26.1	26.2	25.4	25.9	25.2
Pos 05*	35.4	35.6	NDET/NDET	NDET/34.5	33.6	36.2	37.6	37.5	35
Pos 06	27.2	26.7	25	26.9	26.4	27.3	26.8	29.5	26.3
Pos 07	26.3	25.5	22.2	23.3	25.9	26.1	26	28.1	24.7
Pos 08	35.8	34.4	33.6	33	31.7	34.1	35.9	38.5	36.3
Pos 09	18	17.6	15.3	16.4	19.4	19.5	18	19.3	18.6
Pos 10	31.9	32.1	31.1	31.1	31.9	33.6	31.7	34.2	32.2
Pos 11	31.3	31.3	28.1	29.2	30.5	32	31.2	34.6	nd
Pos 12*	37.4	NDET	NDET	NDET	NDET	NDET	NDET/42.6	42.7/NDET	NDET
Pos 13	32.6	33.9	32.5	32.5	NDET	35.7	38.1	40	37.1

a Thirteen negative (Neg) and thirteen positive (Pos) samples were tested. The asterisk indicates that the sample was from a known positive patient in the process of clearing virus.

b NDET, not detected; nd, not done.

## DISCUSSION

This analysis compared the performance characteristics of several *in vitro* diagnostic real-time RT-PCR assays to detect SARS-CoV-2 in high-complexity clinical laboratories in one of the early U.S. epicenters of the COVID-19 pandemic. The results demonstrated excellent performance of a CDC-based LDT and the Cepheid Xpert Xpress, concurring with a previous evaluation that demonstrated high sensitivity of the E-gene and N2 primer sets used by the Cepheid assay (6). The Panther Fusion was somewhat less sensitive than either the LDT or DiaSorin; however, the Panther Fusion detected SARS-CoV-2 RNA in one specimen that was inconclusive (one of two targets detected, thus presumed positive) by the UW CDC LDT. The Roche assay performed on the Cobas 6800 platform detected 28/30 positive samples; both of these discordant specimens had low viral titers (UW CDC LDT *C_T_* of >37), and one was the inconclusive specimen. Therefore, we conclude that all the tested assays show good sensitivity for the detection of SARS-CoV-2, with the UW CDC LDT and Cepheid Xpert Xpress SARS-CoV-2 assays having the best and similar sensitivity, followed by the Roche Cobas 6800, DiaSorin Simplexa, and Panther Fusion SARS-CoV-2 assays.

Our results are chiefly limited by the small sample sets used to compare these different assays as well as asynchronous comparisons that allowed only for pairwise comparisons early in the pandemic. For instance, these asynchronous panels most greatly affected our CDC LDT versus Hologic Panther Fusion comparison, which had a greater proportion of high-*C_T_* positive specimens that resulted in a lower measured sensitivity for the Panther Fusion. In clinical practice, the minor differences in sensitivity are likely to have little effect on Hologic Panther Fusion SARS-CoV-2 assay performance on VTM specimens, given the *C_T_* ranges we have observed in our clinical populations.

Despite their limitations, these data provide a basis for differences in analytical sensitivity at different *C_T_*s that may be seen between platforms. For instance, recent reports have demonstrated a slightly higher analytical sensitivity of the Cepheid Xpert Xpress SARS-CoV-2 assay compared to the Roche Cobas SARS-CoV-2 test, and a slightly lower sensitivity of the DiaSorin Simplexa SARS-CoV-2 assay compared to a modified CDC assay, both of which are concordant with our data (7, 8). We also note that, while analytical sensitivity is of critical importance, many other considerations factor into assay platform selection, including assay availability, cost, turnaround time, and throughput.

Our results provide an early assessment of performance characteristics of five separate assays for the detection of SARS-CoV-2. During March 2020, reagent availability for SARS-COV-2 RT-PCR assays was heavily constrained, necessitating more-limited assay comparisons. All platforms examined here had acceptable performance criteria for testing during the early part of this pandemic. As the supply chain for SARS-CoV-2 RT-PCR attempts to catch up with testing demand, we look forward to additional assay comparison data.
